# Reconfigurable and Efficient Implementation of 16 Boolean Logics and Full‐Adder Functions with Memristor Crossbar for Beyond von Neumann In‐Memory Computing

**DOI:** 10.1002/advs.202200036

**Published:** 2022-03-27

**Authors:** Yujie Song, Xingsheng Wang, Qiwen Wu, Fan Yang, Chengxu Wang, Meiqing Wang, Xiangshui Miao

**Affiliations:** ^1^ School of Optical and Electronic Information Huazhong University of Science and Technology Wuhan 430074 China; ^2^ Hubei Yangtze Memory Laboratories Wuhan 430205 China; ^3^ School of Integrated Circuits Huazhong University of Science and Technology Wuhan 430074 China; ^4^ Wuhan National Laboratory for Optoelectronics Huazhong University of Science and Technology Wuhan 430074 China

**Keywords:** cascade, crosstalk, logic‐in‐memory, low‐power, memristors, reconfiguration

## Abstract

The rise of emerging technologies such as Big Data, the Internet of Things, and artificial intelligence, which requires efficient power schemes, is driving brainstorming in data computing and storage technologies. In this study, merely relying on the fundamental structure of two memristors and a resistor, arbitrary Boolean logic can be reconfigured and calculated in two steps, while no additional voltage sources are needed beyond “±*V*_P_” and 0, and all state reversals are based on memristor set switching. Utilizing the proposed logic scheme in an elegant form of unity structure and minimum cost, the implementation of a 1‐bit adder is demonstrated economically, and a promising circuit scheme for the *N*‐bit adder is exhibited. Some critical issues including the crosstalk problem, energy consumption, and peripheral circuits are further simulated and discussed. Compared with existing works on memristive logic, such methods support building a memristor‐based digital in‐memory calculation system with high functional reconfigurability, simple voltage sources, and low power and area consumption.

## Graphical abstract

This study proposes and successfully verifies by experiments the 16 Boolean logics with a reconfigurable and uniform logic circuit consisted of two memristors and one resistor, implemented in two steps, demonstrating the advantages in speed, power, area, and implementation efficiency. 1‐bit full adder is demonstrated and *N*‐bit full adder scheme is proposed and discussed.

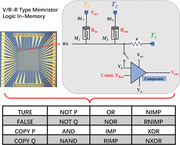

## 1 Introduction

Computation and storage are the two basic functions of an integrated circuit chip. The transfer of data between memory and computing unit often consumes a great deal of power, and many acceleration chips do not resolve well this problem yet.^[^ ^1^, ^2^ ^]^ Accordingly, with computing shifting toward data‐centric, if a novel nonvolatile memory‐based computing structure can be found, the functions of arithmetic and logic unit (ALU) and memory will be fused within a unit (MALU). Thus, the concept of in‐memory computing and potential solutions are proposed, which include logic in‐memory computing (LIM) based on binary devices and neuromorphic computing using multivalue devices. In this solution, data is not required to be saved by a separate memory unit and then calculated by a special ALU. Instead, the storage and calculation operations are conducted directly in this MALU.^[^ ^3^, ^4^ ^]^ From the perspective of computing, computing‐in‐memory can effectively avoid the traditional widespread “memory wall” problem of von Neuman structure, while reconfigurable technology can effectively circumvent the “compile wall” problem as the reconfigurable operators can reconstitute the circuit into a form that most closely resembles the algorithm. The memory fusion technology investigated in this study exploits possibility of the nonvolatile memory and its conditional resistance characteristics to integrate logical operation and information storage, thereby providing fine‐grained support for implementing this architecture‐level memory‐computing fusion effectively.^[^ ^5^, ^6^, ^7^, ^8^, ^9^, ^10^, ^11^, ^12^ ^]^

Memristor turns out to become a strong candidate device for in‐memory computing for its natural nonvolatility, high speed, high density, low power consumption, and full compatibility with CMOS process at low cost.^[^ ^13^, ^14^, ^15^, ^16^ ^]^ Having been proposed and proven already, the LIM of memristors can fall into two categories in principle, that is, stateful logic and nonstateful logic.^[^ ^17^, ^18^, ^19^, ^20^ ^]^ The input and output in the stateful logic are expressed as the resistance state of the memristor. Since 2010, HP Labs has implemented material implication logic functions in a circuit comprising two memristors and a ground resistor.^[14^^]^ Such a scheme facilitates logic cascade and parallel computing. However, with the increase in the logic computing complexity, the number of used devices and the operation complexity increase. Nonstateful logic consists of voltage input and resistance output (*V*–*R*), voltage input and voltage output (*V*–*V*), resistance input and voltage output (*R*–*V*), separately. Specific to *V*–*V* logic and *R*–*V* logic calculation, the output is volatile, and additional storage units are required to store the logic output, which violates the purpose of LIM. Hence, the prospects of these schemes are limited.^[^ ^21^, ^22^, ^23^ ^]^

*R*–*R* logic and *V*–*R* logic are the most promising design schemes at present. *R*–*R* logic calculation is composed of the identical physical form of input and output, resistance, which helps build a simple and pure memristor in‐memory computing system. However, it exhibits some defects (e.g., single logic function, considerable memristor devices, large circuit area, and operation time cost). In traditional *V*–*R* logic calculation, by assigning logical meaning to the operating voltage directly, the process of writing the logical input signal in the memristor device is eliminated. Although this feature can shorten the operation time of logical calculation, in the process of logical iterative calculation, besides calculation, the resistance output of each calculation must be converted into the voltage input of the next calculation, thus increasing the operation time and power consumption, namely the “cascading problem.”^[^ ^24^, ^25^, ^26^, ^27^, ^28^ ^]^

In this study, a *V*/*R*–*R* scheme is proposed to weigh and balance the performance indicators mentioned above. Combining *R*–*R* and *V*–*R* advantages, the *V*/*R*–*R* type solves the cascading problem efficiently (compared with the *V*–*R* type) and significantly reduces the number of devices and operating steps when implementing the complex logic functions (e.g., XOR and XNOR) (compared with the *R*–*R* type). 16 complete Boolean logic functions are altogether implemented in two steps based on two memristors. In this scheme, the input of the binary logic is respectively presented in the form of voltage signal and the resistance state of the memristor. When multiple logic operations are cascaded, the output of the last operation can be directly stored in the memristor, which is used as the input of the next logic operation in the form of resistance. A more detailed comparison of major properties with serial stateful logic is shown in Table S1, Supporting Information. Meanwhile, when the intermediate results need to be transmitted, signal conversion from resistance to voltage can be achieved synchronously within the time step of logical operation. The corresponding reading circuit has been simulated. Last, a 1‐bit full adder based on the LIM structure is implemented, which uses less devices and operation steps than a full adder based on CMOS and most of the existing works based on memristors. Combined with data manipulation issues on memristor crossbars, a promising *N*‐bit parallel full adder circuit scheme is proposed.

## 2 Results and Discussion

### 2.1 Experimental Realization of All 16 Boolean Logic Functions

**Figure 1:**
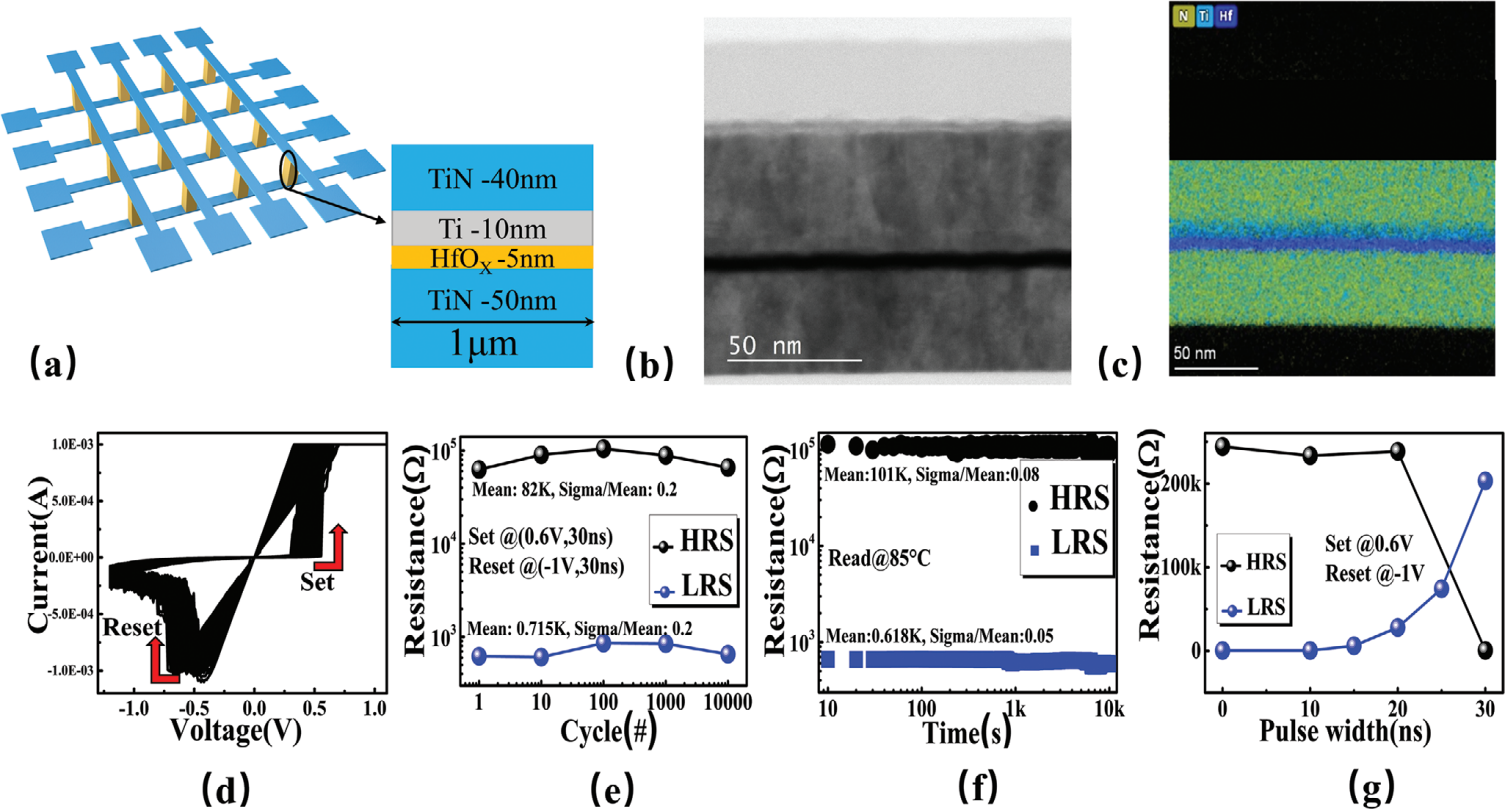
a) The structural diagram of TiN/Ti/HfO*_x_*/TiN memristor. b) High‐resolution TEM image of the cross‐sectional cut of memristor. c) TEM‐EDS mapping images of N, Ti, and Hf in the device. d) Measured resistive switching behavior in dc *I*–*V* sweeping mode. e) More than 10^4^ cycling endurance under voltage pulses (marked with the mean value and sigma/mean for HRS and LRS). f) The retention test result which is up to 10^4^ s at 85 °C (marked with the mean value and sigma/mean for HRS and LRS). g) The switching speed of the memristor.

The memristor device structure used to illustrated the logic scheme is presented in **Figure** **1**a. The hierarchical structure of the device can be characterized under the transmission electron microscope (TEM) with image shown in Figure 1b. The TEM‐EDS mapping images of N, Ti, and Hf for the device are given (Figure 1c). After the 3 V forming voltage initializes the device, 100 consecutive DC cycles are performed (Figure 1d). Thereafter, the set voltage (*V*_set_) and the reset voltage (*V*_reset_) pulses are configured with magnitudes of nearly 0.6 and −1.1 V, respectively. The device switches from a high‐resistance state (HRS) to a low‐resistance state (LRS) when a voltage larger than the set voltage is applied; vice versa, when a voltage magnitude larger than *V*_reset_ voltage is applied on the device, the device changes from LRS to HRS. Exponential test sampling results of 10 000 reversible switches under set (0.6 V, 30 ns) and reset (−1 V, 30 ns) pulses with an HRS‐to‐LRS ratio of 100 are illustrated in Figure 1e. Figure 1f demonstrates that HRS and LRS are nonvolatile maintained for 10^4^ s at 85 °C with a reading bias of 0.1 V. The result of transient switching between the two resistance states is presented in Figure 1g. The device remains at an HRS over 200 kΩ until changed to a ≈400 Ω LRS when pulse width increases to ≈30 ns. According to the test result, for the device at HRS, the resistance drops sharply at a pulse of 0.6 V at 30 ns. Correspondingly, for the device at LRS, the resistance value rises to more than 100 kΩ under a pulse of −1 V in 30 ns. However, only the set operation is employed in the proposed logic scheme.

**Figure 2:**
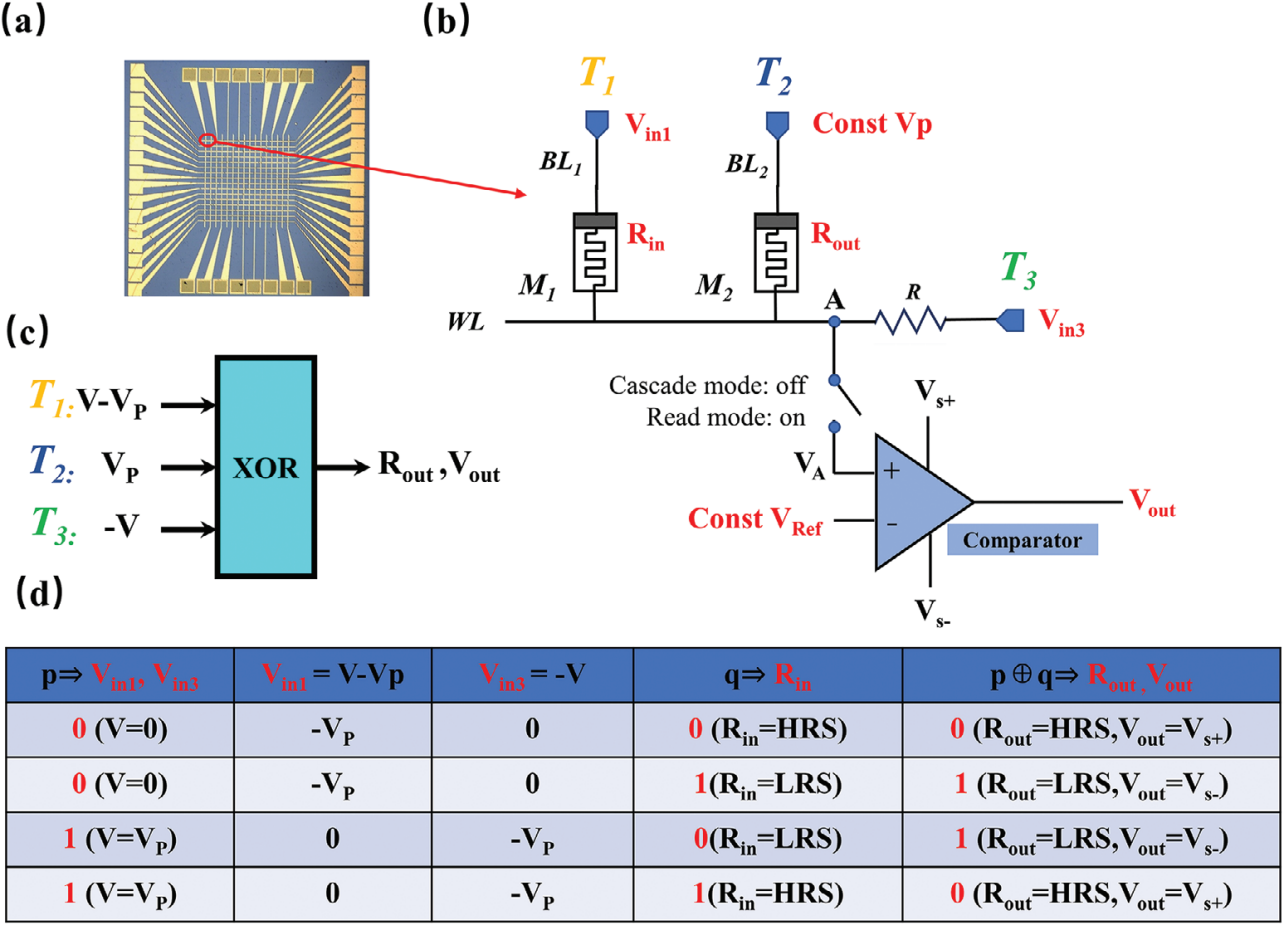
a) Metallographic electron microscopy of the 16 × 16 memristor array. b) The schematic of the memristor‐based LIM circuit. c) The instance diagram of XOR logic gate showing the port configuration. d) The truth table of XOR logic, including the detailed voltage correspondence.

Having the advantages of both *R*–*R* and *V*–*R* types, a novel scheme with extremely uniform kernel of two memristors (M_1_ and M_2_) and one resistor R, is presented for LIM, and it can efficiently implement the complete 16 Boolean logics by configuring the particular biases on terminals. The two inputs of a given logic caculation are either encoded with logic configuration into the corresponding voltage applied upon the first memristor or the resistance state of the first memristor. A 16 × 16 array composed of the above memristors is used to test the 16 Boolean logic schemes and 1‐bit fuller adder (**Figure** **2**a). A Boolean logic circuit, illustrated in Figure 2b, comprises the logic kernel, a switch, and a comparator. The circuit has two working modes. When the switch is off, it works in cascaded mode. That is, the output is a resistance state (*R*_out_). The next logic operation can be carried out directly without signal transformation. When the switch is on, the comparator is connected to the circuit, and the logic output result is achieved by comparing the voltage level of node A with the fixed reference value. The R acts not only as a voltage divider but also as a sampling resistor of the reading circuit coincidentally. A proper load resistor serially connected with memristor has been reported to improve the switching uniformity and endurance of the memristor.^[^ ^29^, ^30^, ^31^ ^]^ The value of R is set to $R=\sqrt{R_{H}\cdot R_{L}}$ which depends on the memristors parameters. The resistance state (*R*_out_) of M_2_ and the voltage value of the output of the comparator (*R*_out_) both stand for the result of logic calculation in reading mode. In our work, the HRS and *V*_s+_ correspond to the logical value “0,” and the LRS and *V*_s−_ represents the logical value “1.” The control terminals T_1_, T_2_, and T_3_ are, respectively, connected to the positive pole of M_1_, the positive pole of M_2_, and one end of R. The negative poles of M_1_, M_2_, and the other end of R are linked on the identical word line. The logic implementation depends on the voltage distribution among the devices under applied voltages. T_1_, T_2_, and T_3_ only need to configure three types of voltages (±*V*_p_, 0) to achieve 16 Boolean logic. *V*_p_ denotes a constant that should satisfy *V*_set_/2 < *V*_p_ < *V*_set_, so the M_2_ initially in HRS cannot switch to the LRS when the voltage drop does not reach *V*_set_. Moreover, the resistance state of the M_2_ should be reversed when the voltage across the memristor exceeds 2*V*_p_. In addition, *V*_p_ < 2|*V*_reset_| is required. Thus, the memristor originally in LRS will maintain its current state during the operation. *V*_p_ is set at 0.4 V as pulse amplitude, and pulse period is set at 50 ns in subsequent simulations and tests.

Take exclusive OR (XOR) logic as an example to illustrate the logical calculation process. A block diagram (Figure 2c) shows the ports configuration of the proposed three‐input XOR gate structure. Figure 2d represents XOR logic's truth table, indicating the mapping relationship between logical input/output and physical quantities of voltage and resistance. *V* represents a variable determined by the logic input *p*. Logic value “0” represents *V* = 0; logic value “1” corresponds to *V* = *V*_p_. Therefore, the *V*_in1_ and *V*_in3_ configured to terminals T_1_ and T_3_ are determined by the logical input “*p*.” Another input *q* is mapped as the resistance state of M_1_ (*R*_in_). It is noteworthy that the original data is undamaged and still stored in M_1_ after the calculation, which helps maintain the integrity of the original data.

The XOR gate is implemented in one step of writing, one step of logic operation:

Step 1: The default initial state of memristors is HRS. The second memristor (M_2_) keep HRS, and the resistance state corresponding to input *q* is written to the first memristor (M_1_);
Step 2: Apply corresponding voltage pulses to the control terminals T_1_, T_2_, and T_3_, respectively (T_2_ is set as a constant value of *V*_p_ regardless of the input). The switch is set to ON when the calculation result needs to be read out, that is, the output is transmitted in the form of voltage.

**Figure 3:**
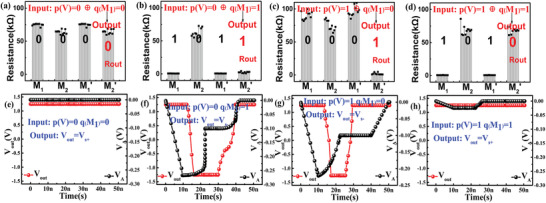
a−d) Experimental results for the four possible input−output combination of XOR logic. e–h) Simulation results for the four possible input–output combination of XOR logic in read mode.

According to the truth table of XOR logic, **Figure** **3**a−d presents the resistance transitions measured in the actual circuit under four input conditions. Each condition has been tested ten times. The feasibility of the read mode is verified by involving the read circuit at the time step of a logical operation (Step 2). The rise, fall time, and the width of input pulse for T_1_, T_2_, and T_3_ in simulation both are 10, 10, and 30 ns. In addition to the *V*_out_, the node V_A_, sampled for comparison with a fixed reference voltage, is also shown in Figure 3e,f. When *V*_A_ > *V*_Ref_, *V*_out_ = *V*_s+_, the output of logic operation is “0.” Vice versa, when *V*_A_ < *V*_Ref_, *V*_out_ = *V*_s−_, the output is “1.”

**Figure 4:**
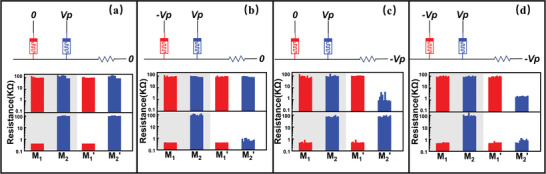
a–d) Experimental results for the eight possible input–output combination of logic scheme.

Based on the theoretical calculation from Ohm's law and Kirchhoff's law, we analyze that by arranging and combining variables at T_1_ and T_3_ terminals with the resistance state of M_1_, port configuration schemes of 16 Boolean logic can be achieved. There are four scenarios of configurable voltages at T_1_ and T_3_ with each terminal selected from {−Vp, 0} set, and two possiblilities of M_1_ resistance input variable *q*, thus there are eight input conditions. The circuit diagrams in **Figure** **4** enumerate the possible voltage combinations at the control end. The upper and lower bars of the histograms show two scenarios when the input resistance of M_1_ is HRS and LRS, respectively. Eight possible scenarios are demonstrated and verified. Consistent with the truth table of a certain logic function, four corresponding actual calculation scenarios can be selected from the mentioned eight scenarios, and then the corresponding port configuration is given from the set of 0, −*V*, −*V*_p_, and *V* − *V*_p_. XOR logic is taken as an example again. When the input *p* = 0, *q* = 0, the output state of *R*_out_ = 0, M_2_ should maintain a high impedance state. Meantime, the input resistance state of M_1_ is HRS (>> R) after Step 1, thereby concluding that the voltage of the control terminal T_3_ in the operation step should be 0. When the input *p* = 0, *q* = 1, and the output state of *R*_out_ = 1, M_2_ should be transformed to LRS. The state of M_1_ is LRS, less than the resistor R, which reveals that the voltage of the control terminal T_1_ in the operation step should be −*V*_p_. When the input *p* = 1, *q* = 0, and the output state of *R*_out_ = 1, M_2_ should switch to LRS. In this case, the resistance state of M_1_ is HRS, suggesting that the voltage of the control terminal T_3_ in the operation step should be −*V*_p_ to ensure enough voltage drop. According to the truth table, when the input *p* = 1, *q* = 1, and the output state of *R*_out_ = 0, M_2_ should maintain HRS, the state of M_1_ is LRS after Step 1, and the control terminal T_1_ in Step 2 should be 0. Combining the above four conditions, it can be concluded that: when the input *p* = 0, T_1_ = −*V*_p_ and T_3_ = 0; when the input *p* = 1, T_1_ = 0 and T_3_ = −*V*_p_. Accordingly, the port signal of the control terminals T_1_ and T_3_ are *V* − *V*_p_, −V, respectively. Depending on the mentioned infer processing, the port configurations of control terminals corresponding to 16 logic functions are deduced with effect (**Table** **1**).

**Table 1:** 16 Boolean logics’ port configurations for control terminal T_1_ and T_3_

Logic function	T_1_	T_3_
TURE	−*V*_p_	−*V*_p_
FALSE	0	0
COPY *P*	−*V*	−*V*
COPY *Q*	−*V*_p_	0
NOT *P*	*V*−*V*_p_	*V*−*V*_p_
NOT *Q*	0	−*V*_p_
AND	−*V*	0
NAND	*V*−*V*_p_	−*V*_p_
OR	−*V*_p_	−*V*
NOR	0	*V*−*V*_p_
IMP	−*V*_p_	*V*−*V*_p_
RIMP	−*V*	−*V*_p_
NIMP	0	−*V*
RNIMP	*V*−*V*_p_	0
XOR	*V*−*V*_p_	−*V*
NXOR	−*V*	*V*−*V*_p_

### 2.2 Implementation of 1‐bit Full Adder

Besides Boolean logic computing, arithmetic computing also serves an essential role in ALU. In the CMOS configuration, arithmetic functions are constructed with many Boolean logic gates. This can be implemented in the MALU, where the combinational functions are realized through sequential logic functions. Full adder is the most common and practical form of combinatorial logic.^[^ ^32^, ^33^, ^34^, ^35^, ^36^ ^]^ A 1‐bit full adder can be realized based on six devices in six steps through the abovementioned logic circuit. For a 1‐bit binary full adder, there are three inputs (i.e., addend *a*_i_, summand *b_i_*, and carry‐in *c_i_*) and two outputs (i.e., summary *s_i_* and carry‐out *c*_(_ *_i_*_+1)_). Furthermore, the output results can be written as:

(1) $$s_{i}=a_{i}\;⊕b_{i}⊕c_{i}$$

(2) $$c_{i+1}=a_{i}\;\cdot b_{i}+\left(a_{i}⊕b_{i}\right)\cdot c_{i}$$

**Figure 5:**
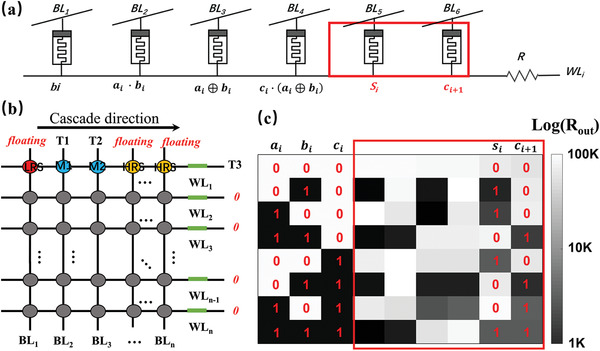
a) The circuit diagram of 1‐bit full adder. b) Crossbar schematic marked with clamp voltage used for one‐full adder. c) Experimental results for eight logic input conditions (including intermediate results).

**Figure** **5**a illustrates the circuit of a 1‐bit full adder, in which six adjacent cells are selected on a row or column in the crossbar. Cascade computing is demonstrated on the memristors. The calculation results are stored in M_5_ and M_6_. The calculation steps in **Table** **2** clarified the voltage configuration corresponding to each terminal in each step. Practical issues arose regarding data manipulation in a large‐scale array. Considering the crosstalk problem, the biasing voltage setting of the memristor not involved in the calculation must be considered. First, the biasing voltage is selected from the typical *V*/2, *V*/3, gnd‐floating, gnd‐gnd, and floating‐gnd schemes.^[^ ^32^, ^33^ ^]^ Gnd‐floating is referred to the scheme with grounded unselected word lines and floating unselected bit lines and so forth. According to the superposition theorem, when the two ends of the noncalculated memristors in HRS are clamped at *V*_p_/2 or *V*_p_/3, the potential of the bottom electrode of the calculated line (WL) will be raised. With the increase of array size, it will approach *V*_p_/2 (*V*_p_/3) which leads to insufficient voltage drop when M_2_ needs overturned, resulting in calculation errors. In addition, the calculated memristors along the direction of the cascade can save “0” and “1;” when the storage information is “1” the calculated memristors are at LRS. It is not difficult to infer that the clamp voltage of this type of memristors can be directly loaded to the bottom electrode of the calculation line (WL) through the low resistance memristor. After analysis, to ensure the accuracy of the calculation, the biasing voltage scheme has been determined to use gnd‐floating (Figure 5b). The above analysis has been verified by simulation combining the conclusions of existing work.^[^ ^33^ ^]^ It is also found that properly trimming the voltage value at T_2_ control end can improve the calculation accuracy inside (*V*_set_/2, *V*_set_). Unlike the bottom electrode connected to the resistance network and affected by other cells, the top electrode is independent without voltage drop loss. Appropriate adjustment can make up for the voltage loss of the bottom electrode of M_2_.

**Table 2:** Detailed operation steps and voltage configuration in the addition function

STEP	Operation	BL_1_	BL_2_	BL_3_	BL_4_	BL_5_	BL_6_	WL_i_
1	Write in *b_i_*	Gnd (*b_i_* = 0); *V*_p_ (*b_i_* = 1)	Gnd	Gnd	Gnd	Gnd	Gnd	Gnd (*b_i_* = 0); −*V*_p_ (*b_i_* = 1)
2	*a_i_* · *b_i_*	0 (*a_i_* = 0); ‐*V*_p_ (*a_i_* = 1)	*V*_P_	Gnd	Gnd	Gnd	Gnd	Gnd
3	*a_i_* ⊕*b_i_*	− *V*_p_ (*a_i_* = 0); 0 (*a_i_* = 1)	floating	*V*_p_	Gnd	Gnd	Gnd	0 (*a_i_* = 0); ‐*V*_p_ (*a_i_* = 1)
4	*c_i_* · (*a_i_* ⊕*b_i_*)	floating	floating	0 (*c*_i_ = 0); ‐*V*_p_ (*c*_*i*_= 1)	*V*_p_	Gnd	Gnd	Gnd
5	*c_i_* ⊕(*a_i_* ⊕*b_i_*) (S*_i_*)	floating	floating	‐Vp(*c_i_* = 0) 0 (*c_i_* = 1)	floating	*V*_p_	Gnd	0 (*c_i_* = 0) ‐Vp (*c_i_* = 1)
6	*a_i_* · *b_i_* + (*a_i_* ⊕*b_i_*) · *c_i_* (*c*_(_ *_i_*_+1)_)	floating	floating	floating	floating	−*V*_p_	*V*_p_	0 (*a_i_* · *b_i_* = 0) ‐Vp (*a_i_* · *b_i_* = 1)

Figure 5c presents the experimental test results of eight possible inputs of a 1‐bit full adder. In the experimental test, the results which are consistent with the truth table are successfully obtained. Three columns on the left in Figure 5c are three inputs, while the six columns on the right represent the six devices in the 1‐bit full adder circuit. After the calculation, the resistance value in Figure 5c is the state of each memristor. It is noteworthy that the logic implementation proposed in this study is nondestructive, which reveals that the resistance state of the memristor as the input remains unchanged after the logic operation is completed. These features will reduce a deal of backup process and lay great convenience for extending the logic circuit to other applications. In the application of a 1‐bit full adder, *a_i_* ⊕b_*i*_ and *a_i_* · b_*i*_ can be calculated by multiplexing the b_*i*_ written in memristor M_1_, thereby saving more hardware consumption for the overall calculation. When the logic circuit proposed in this study is applied to a larger system with a multistep logic calculation, it has been demonstrated to outperform other methods to a certain extent. If the logic calculation of the current step should use the result of the previous step, the characteristic of the circuit that does not damage the written data in the calculation makes the logic cascading easy to realize.

### 2.3 A Feasible Circuit Architecture for *N*‐bit Adder

**Figure 6:**
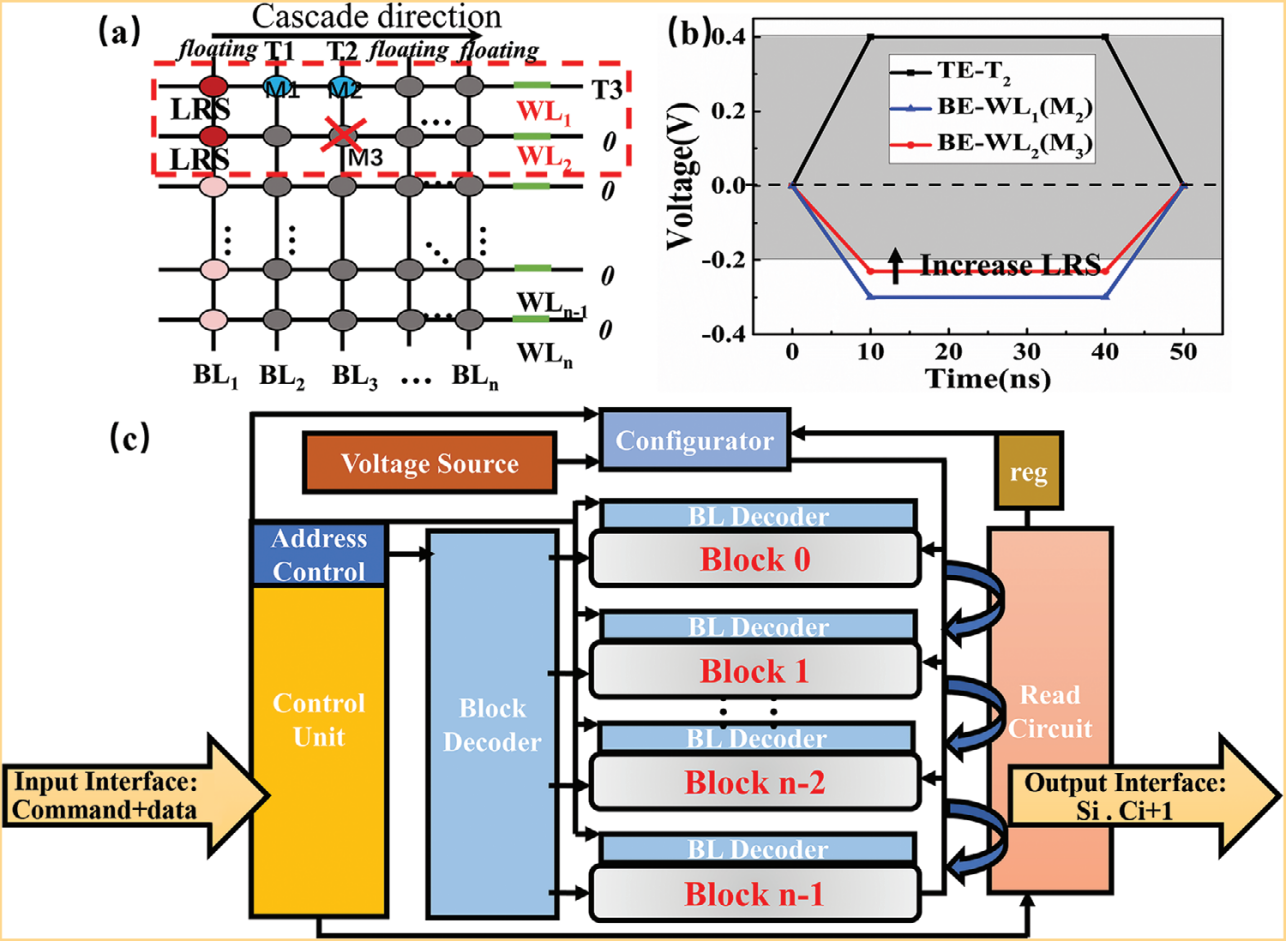
a) The mis‐operation issue caused by the bitwise parallel operation of memristor crossbar array, the mis‐operated M_3_ is marked cross. b) Simulation results of mis‐operation conditions. c) A feasible circuit architecture for *N*‐bit adder.

Improving parallelism can maximize the use of computing resources. To increase the computational parallelism, the data manipulation of the *N*‐bit adder on the memristor array relies on the bitwise parallelism and blockwise parallelism.^[^ ^34^, ^37^, ^38^ ^]^ For bitwise parallel computing, the operands must be aligned before the operation, making the data manipulation more important. As can be inferred from the previous section, each line corresponds to a 1‐bit adder, so the *N*‐bit adder needs an n‐line parallel operation. However, it is found that the data mapping based on memristor crossbar array inevitably mis‐operate other cells. **Figure** **6**a shows that the cells of noncalculated lines can be possibly mis‐operated when there are more than two LRS in the written data or calculated cells on the same BL. Figure 6b explains that the M_3_’s voltage drops beyond *V*_set_ simultaneously for the reason of two LRS memristors on the same BL_1_ in front when the voltage drop between M_2_’s control ends exceed *V*_set_. Increasing the device LRS parameter can effectively mitigate this problem. However, this issue cannot be wiped out from root based on the crossbar array.

Therefore, we focus on block parallelism to design an *N*‐bit full adder scheme based on the memristor crossbar array. In the proposed circuit architecture, arrays of memristive switches are dedicated to performing 1‐bit or specific functions. These arrays are defined as function blocks. The BL of each block can be selected uniformly for parallel operation in one clock cycle or separately for serial manner. Specific to the implementation of serial carry *N*‐bit adder, the steps marked in blue in Table 2 need to be executed serially from the algorithm, for those steps involving carry must wait for the last bit to start the calculation. Besides, the implementation of computing in‐memory needs the assistance of external circuits, including control unit (CU), that are responsible for the instruction decoding and determining the state trend of the whole system. The CU selects the corresponding block according to whether the command indicates the parallel mode or the serial mode. Each block selects the corresponding two BLs following the address control. It is worthy of mentioning that the number of memristors executing arbitrary logic in the recommended scheme is fixed and can be cascaded, that is, the address control is simple. For the addressing circuit, either direct or counter address can continuously increases one to find the next memristor because of the seamless cascading. After selecting the cells to be calculated, the configurator chooses the voltage source and loads it on the block on the basis of the Table 1. When it is necessary to perform the signal conversion such as transmitting carry signal, the read circuit can be involved with the on‐state switch. The output *V*_out_ is temporarily stored in the register. The main function of the register is to store the data represented by voltage signals for the proposed scheme involving both voltage and resistance signals. Using AND, OR, and XOR gates of our scheme, this *N*‐bit adder based on crossbar array required 3N + 3 clocks and 6N memristors. In contrast with LIM based on memristors, it has been calculated that the energy consumption required to calculate XOR logic is no more than 1 pJ, which is competitive.^[^ ^39^ ^]^ Compared with CMOS circuits, the area of the crossbar array can be significantly reduced, and the consumption of static power and data handling can be eliminated (Table S2, Supporting Information). It is suitable for the application scenario of edge processors, due to the fact that power consumption is a very important consideration in the edge computing and design.^[^ ^40^, ^41^, ^42^ ^]^

## 3 Conclusion

In brief, this study proposes and experimentally demonstrates that two memristors can achieve the calculation of 16 Boolean logic functions in two steps by adopting *V*/*R*–*R* setting. A prototype MALU is verified. Owing to the unity of memory and computation, the proposed MALU can exhibit high energy‐efficiency cause of the near‐zero static power consumption feature and reduction in data access. The fundamental circuit is based on a memristive device with a Ti/HfO*_x_*/TiN structure exploiting its sufficiently large switching ratio and extremely fast resistance‐state switching speed to improve the calculation accuracy and overall energy consumption. A 1‐bit full adder based on the logic circuit is constructed, highlighting the advantages of easy cascading, the superiority of the logic scheme, and providing inspiration for subsequent research on in‐memory computing. The inevitable crosstalk problem on the memristor crossbar array is also analyzed in detail. Finally, a complete design idea of the *N*‐bit serial carry adder using block parallelism is given. We believe this work could be a meaningful step forward to building a practical memristor‐based processor for in‐memory computing.

## 4 Experimental Section

### Device Fabrication

The logic function was demonstrated with the 1 × 1 µm^2^ TiN/Ti/HfO*_x_*/TiN memristor. First, TiN bottom electrode was fabricated by atomic layer deposition (ALD). Then, a 5‐nm HfO*_x_* layer was deposited by ALD. Ti top electrode was fabricated by sputtering on the functional layer. Last, 40‐nm TiN was deposited on the top electrode to avoid the oxidation of Ti.

### Measurement

All electrical measurements were performed using Key B1530A semiconductor parameter analyzer. The resistor was additionally placed between the array and the source measurement unit. The simulation was performed by Virtuoso using a binary electrical behavior model for memristors.

### Statistical Analysis

For the data listed, to ensure the representative of the sample data, statistical tests were performed on more than 50 devices in total, and more than 50 DC and pulses were tested for each device. The sample size, mean value, and the ratio of standard deviation and mean for each group data were explained in the corresponding position and were checked for differences, with the significance level (alpha value) taken as 0.05. More specifically, the statistical results of *V*_set_ were critical in the setting of *V*_p_. For the reason that *V*_p_ needs to satisfy (*V*_set_/2, *V*_set_), the statistical evaluation of *V*_set_ was important for the accuracy of the logical calculation. To demonstrate the critical statistical data in relation to the set logic operation, Figure S1, Supporting Information, showed the *V*_set_ statistics of 12 randomly selected memristors from the array with 50 cycles per device. By two‐sided hypothesis test, the authors assumed that H_0_:*V*_set_ would fall outside the (0.5 and 0.7 V) interval first. If H_0_ was true, the *P* value was 0.1 (select the device's data that occurs H_0_ most). Since the *P* value was greater than 0.05, the H_0_ hypothesis was accepted. Next, it was assumed that H_1_: *V*_set_ would fall outside the (0.4 and 0.8 V) interval. In this case, H_1_ was true only once (it was eliminated as an outlier). So, test statistics yield *P* = 0.02 which was smaller than 0.05, Thus, the authors rejected the H_1_ hypothesis. The statistical range of *V*_set_ was determined at (0.4 and 0.8 V) through above statistics analysis.

## Conflict of Interest

The authors declare no conflict of interest.

## Supporting information

Supporting Information

## Data Availability

The data that support the findings of this study are available from the corresponding author upon reasonable request.
