# Supplementary material for: Reconfigurable and Efficient Implementation of 16 Boolean Logics and Full‐Adder Functions with Memristor Crossbar for Beyond von Neumann In‐Memory Computing
Source: Adv Sci (Weinh). 2022 Mar 27;9(15):2200036. doi: 10.1002/advs.202200036 (PMC9130921; doi:10.1002/advs.202200036)
Supplement: Supplementary file 1 — Supporting Information [file ADVS-9-2200036-s001.pdf]

ADVANCED SCIENCE
Open Access

Supporting Information

for *Adv. Sci.*, DOI 10.1002/advs.202200036

Reconfigurable and Efficient Implementation of 16 Boolean Logics and Full-Adder Functions with Memristor Crossbar for Beyond von Neumann In-Memory Computing

Yujie Song, Xingsheng Wang, Qiwen Wu, Fan Yang, Chengxu Wang, Meiqing Wang and Xiangshui Miao*

Supporting Information

Reconfigurable and Efficient Implementation of 16 Boolean Logics and Full-Adder Functions with Memristor Crossbar for Beyond von Neumann In-Memory Computing

Yujie Song, Xingsheng Wang, Qiwen Wu, Fan Yang, Chengxu Wang, Meiqing Wang, Xiangshui Miao*

Y.J. Song, Prof. X.S. Wang, Q.W. Wu, F. Yang, C.X. Wang, M.Q. Wang, Prof. X.-S. Miao are with School of Optical and Electronic Information, Huazhong University of Science and Technology, Wuhan, 430074, China.
Prof. X.S. Wang and Prof. X.-S. Miao are also with Hubei Yangtze Memory Laboratories, Wuhan 430205, China; School of Integrated Circuits, Huazhong University of Science and Technology, Wuhan, 430074, China; Wuhan National Laboratory for Optoelectronics, Huazhong University of Science and Technology, Wuhan, 430074, China.
E-mail: xswang@hust.edu.cn

Table S1. Comparison of major properties of the sequential memristive logic design concepts.

	IMPLY [1-5]	MAGIC [6]	CMOS-like [7]	Our Work
**Computation**	sequential	sequential	sequential	sequential
**Logic state variable**	memristance	memristance	voltage	Memristance +voltage
**Main circuit structure**	Memristors + resistor	memristors	memristors	Memristors + resistor
**Implement any 16 Boolean logic**	Yes	Difficult	Difficult	Yes
**Number of devices for different logic**	Not fixed	Not fixed	Not fixed	Fixed
**Cascading logic gates**	Possible	Difficult	Not possible	Yes
**Complexity of driving/auxiliary circuitry**	High	Very high	Very high	Medium

A comparison with other influential sequential stateful logic schemes is shown in **Table S1**. Among the sequential processing circuit design concepts, the MAGIC [6] and the CMOS-like [7] schemes require a much higher complexity for their driving circuitry, although those

improve significantly the computation time of IMPLY [1-5] logic. The IMPLY logic assumes a relatively simple circuit structure and can realize 16 Boolean logic through logical cascade compared with MAGIC logic and CMOS-like type. However, this scheme requires a different number of devices for different logical operations, and to realize the more complex calculation, the more devices are needed. In our scheme, the number of memristors to realize any type of logical computing circuit is two, and the number of calculation steps is fixed, which obviously reduces the difficulty of control circuit design.

Table S2. Device number, latency, power consumption, and energy consumption comparison of XOR logic circuit between CMOS technologies and memristor-based.

	Device numbers	Latency	Power consumption	Energy consumption
**CMOS [8]**	4	1.43ns	32mW	Dynamic + static power
**Memristor (Our work)**	2	50ns	11μW	Dynamic power

From the perspective of the underlying basic circuit, **Table S2** is the comparison between the one-bit XOR circuit under the same μm -level process node (@CMOSTSMC 0.8-μm) and the proposed scheme[8]. It can be seen that the delay of the CMOS circuit is better than that of the memristor, and the performance of XOR circuit based on the memristor is better than that of CMOS in terms of circuit area and power consumption. What's more, the memristor-based circuit can eliminate the static power consumption caused by the leakage of transistors.

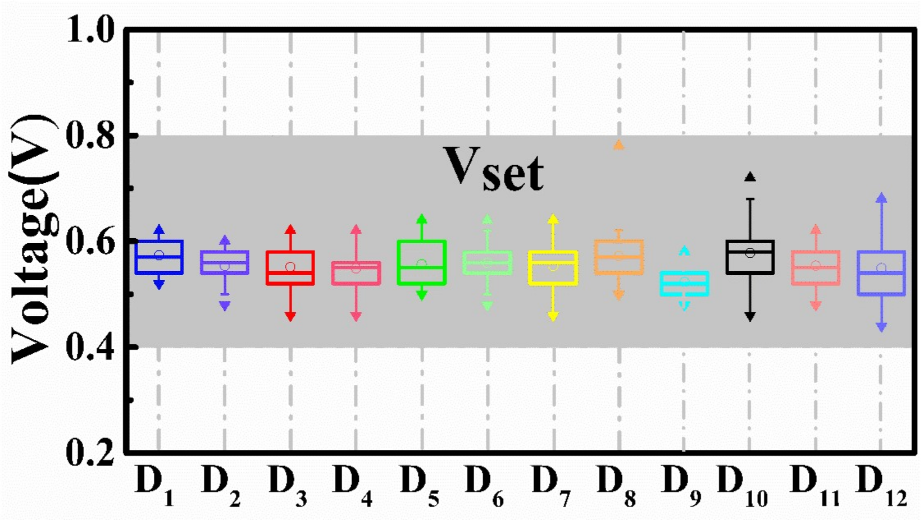

Figure S1. The V_{set} statistics of 12 randomly selected memristors from the array with 50 cycle measurements per device.

[1] J. Borghetti, G. S. Snider, P. J. Kuekes, J. J. Yang, D. R. Stewart, R. S. Williams, *Nature*. **2010**, 464, 873.

[2] T. Dalgaty, N. Castellani, C. Turck, K.E. Harabi, E. Vianello, *Nature.*, **2021**, 4, 151.

[3] I. Vourkas, G. C. Sirakoulis, *IEEE Circuits Syst. Mag.* **2016**, 16, 15.

[4] S. Kvatinsky, G. Satat, N. Wald, E. G. Friedman, A. Kolodny, U. C. Weiser, *IEEE Trans. VLSI Syst*, **2014**, 22, 2054.

[5] M. Maestro-Izquierdo, J. Martin-Martinez, A. C. Yepes, M. Escudero, R. Rodriguez, M. Nafria, A. Xavier, A. Rubio, *IEEE Trans. Emerging Topics Comput.* **2017**, 7, 545.

[6] S. Kvatinsky, D. Belousov, S. Liman, G. Satat, U. C. Weiser, *IEEE Trans. Circuits Syst*, **2014**, 61, 895.

[7] I. Vourkas, G. C. Sirakoulis, *Microelectron*, **2014**, 45, 59.

[8] J.-M. Wang, S.-C. Fang, and W.-S. Feng, *IEEE J. Solid-State Circuits.* **1994**, 29, 780.
